# Pattern of ultrasonographic findings of disorders of the ankle joint complex in patients presenting with ankle pain at the department of diagnostic imaging, university of Nairobi

**DOI:** 10.11604/pamj.2018.31.116.14049

**Published:** 2018-10-17

**Authors:** Muhammad Omar Bashaeb, Timothy Musila Mutala, Ian Mathenge Muriithi

**Affiliations:** 1 Department of Diagnostic Imaging and Radiation Medicine, University of Nairobi, Nairobi, Kenya

**Keywords:** Musculoskeletal, ultrasound, ankle, pain

## Abstract

**Introduction:**

the ankle joint is a frequently injured joint. It is also affected by inflammatory, infectious and tumoral lesions. Ultrasound is a safe and cost-effective imaging tool when evaluating the ankle joint, as most structures are superficial and accessible. The aim of the study was to determine the pattern of ultrasonographic findings of disorders of the ankle joint complex in patients presenting with ankle pain.

**Methods:**

a total of 43 consecutive patients with ankle pain referred for an ankle radiograph or ankle ultrasound were examined. Statistical analysis was done using SPSS version 20.

**Results:**

abnormalities were found in 60% of the examinations performed. The abnormalities were more common in female patients accounting for 61.4% of the abnormalities detected. The most common finding was synovial hypertrophy seen in 26% of the patients.

**Conclusion:**

ankle ultrasound was able to identify a large number of pathologies. This demonstrates the usefulness of ultrasound in the evaluation of a patient with ankle pain.

## Introduction

The use of musculoskeletal ultrasound by radiologists has taken a back seat not only in Kenya but also in the developed world. The reason for this is that many believe musculoskeletal ultrasound is difficult and has a steep learning curve [1]. Most radiology residency programs do not incorporate musculoskeletal sonography in their curriculums, hence the reluctance of radiologists in performing the examination [1]. Another aspect is the higher remunerations involved in reading Magnetic Resonance Imaging (MRI) examinations as compared to performing sonography [1] leading to the preferential use of MRI over sonography. This has resulted to the increasing use of musculoskeletal ultrasound by non-radiologists. It has been shown that between the period of 2000 and 2009, in the United States, 71.8% of ultrasounds done in the office setting were done by non-radiologists [2]. Preference is given to MRI because it has more standardized protocols, its high spatial resolution, wide field of view and its ease of interpretation [1]. While these statements are largely true, in experienced hands musculoskeletal ultrasound has shown to be a complementary or even an alternative to MRI examinations [1]. Ultrasound is a cost-effective, readily available imaging modality. Its added advantages include; it is patient friendly, allows for dynamic evaluation and facilitates bilateral comparison. Physiologic information can also be provided by the use of color and power Doppler [1]. It can also be used in guiding therapeutic interventions. One of its disadvantages is its lack of ability to interrogate deeper structures. With recent advances, ultrasound probes can interrogate superficial structures with better resolution. A 15MHz probe gives a resolution of 200μm, greater than routine MRI [3]. The ankle joint is the most frequently injured joint, and it commonly involves the lateral compartment [4]. Although most injuries are treated conservatively, adequate assessment of injuries is required. Most of the structures that comprise the joint are superficial and easily identifiable and accessible to ultrasound [5]. Ultrasound has been shown to be useful in detecting injuries in patients with acute ankle injury, or in patients who present with symptoms of chronic ankle pain [6]. Ankle sprains represent 5% of all casualty consultations in the United Kingdom [7].

The ankle joint is initially imaged using plain radiography with the antero-posterior, the mortise view and lateral views. Radiography evaluates the bony elements of the joint but does not interrogate the soft tissue structures around it. A normal radiograph of the ankle joint does not completely rule out injury to the surrounding soft tissue structures [8]. The ankle joint is surrounded by tendons, blood vessels, ligaments and nerves which need to be evaluated in a patient presenting with ankle pain. Further evaluation is usually performed by MRI [8]. While MRI is excellent for assessing musculoskeletal disorders it is an expensive imaging modality [9]. The ankle joint can be affected by traumatic injuries, inflammatory conditions, instability and osteoarthritis [10]. Traumatic injuries could involve fractures or ligamentous injuries. The most common cause of ankle pain results from an ankle sprain [10]. This is more evident in sports related injuries [11]. Ultrasound has been shown to have high accuracy rates in detecting ankle tendon and ligamentous tears. In a study by Rockett *et al.* [12], ultrasound was found to be more accurate and sensitive than MRI in diagnosis of ankle tendon tears. Ultrasound showed a sensitivity of 100% and accuracy of 94.4% while MRI showed a sensitivity of 23.4% and an accuracy of 65.75%. Grant *et al*. [13] correlated the findings of peroneal tendon tears with surgical findings. Results revealed ultrasound had 100% sensitivity, 85% specificity and 90% accuracy in diagnosing peroneal tendon tears. Friedrich *et al*. [14] evaluated the lateral aspect of traumatized ankles and showed that in cases involving the anterior talofibular ligament, ultrasound correlated with operative findings in 100% of the cases and in 92% of cases involving the calcaneofibular ligament. Locally, patients presenting with ankle pain are often imaged with plain radiography. The next imaging modality used is MRI. Ultrasound is capable of being an alternative to the more expensive option of MRI. It is cheaper and readily available in most regional hospitals. It is also able to visualize the soft tissue structures around the ankle joint and give a diagnosis with high sensitivity, specificity and accuracy. The aim of this study is to demonstrate the usefulness of sonography in evaluating the ankle joint and by extension increase the awareness of sonography as an alternative imaging modality when evaluating joints in general.

## Methods

This study was a cross-sectional study and was conducted at the Department of Diagnostic Imaging, University of Nairobi between August 2016 and May 2017. It included 43 patients. Ethics approval was obtained from the Kenyatta National Hospital/University of Nairobi ethics and research committee.

**Study population:** all patients who attended the Department of Diagnostic Imaging, University of Nairobi for an ankle radiograph in which no fractures were seen and patients referred for an ankle ultrasound.

**Inclusion criteria:** patients referred for an ankle radiograph; patients referred for an ankle ultrasound; patients above 18 years of age

**Exclusion criteria:** patients with acute lower limb fractures; patients who declined consent to the examination

**Sampling method:** the sampling method used was a convenient sampling method.

**Materials and equipment:** consecutive patients who were referred to the Department of Diagnostic Imaging, University of Nairobi, and fit the inclusion criteria were included in the study. After informed consent had been obtained, the biodata and clinical history were taken and filled using a structured questionnaire by the principal investigator. The US machine used was either a General Electric Logic 7 or a Philips HD 11. A linear transducer was used with a frequency of 12MHz. The ankle ultrasound was then performed according to the standard protocol by the European Society of Musculoskeletal Radiology technical guidelines VI. The examinations were performed by the principal investigator and confirmed by my co-authors who are consultant radiologists. The ultrasound was done at no extra charge to the patient. Evaluation of the anterior, lateral, medial and posterior compartments was performed as follows: *anterior compartment:* the patient was placed supine with the knee flexed and foot placed flat on the examination couch. The extensor tendons and the tibiotalar joint were then evaluated; *lateral compartment:* the foot was inverted with the patient supine and the lateral collateral ligaments and the peroneal tendons were examined; *Medial compartment:* the patient was placed in a frog-lateral position. The flexor tendons and the medial collateral ligament were evaluated; *posterior compartment:* the patient was placed in a prone position with the foot hanging over the couch. The Achilles tendon and the plantar fascia were examined. Imaging diagnostic criteria were used to classify and diagnose the pathology. The criteria used are described below: *tendinopathy:* loss of the normal fibrillary echotexture of the tendon; *tenosynovitis:* hypoechoic thickened tissue with or without fluid within the tendon sheath which may exhibit Doppler signal; *partial tendon tear:* partial thickness interruption of the tendon fibres; *tendon complete tear:* full thickness interruption of the tendon fibres; *plantar fasciitis:* hypoechoic thickened plantar fascia >4mm; *partial ligament tear:* thickened inhomogeneous irregular ligament with minimal discontinuity; *full thickness Ligament tear:* full thickness discontinuous tendon with retraction of the fibres; *synovial hypertrophy:* abnormal hypo-echoic to subdermal fat (may be iso-hyperechoic) intra-articular tissue that is non displaceable and poorly compressible and which may exhibit Doppler signal; *joint effusion:* abnormal hypoechoic intra-articular material that is displaceable and compressible; *Joint Erosion:* an intra-articular discontinuity of the bone surface. Abnormalities were categorized as tendon, joint, ligamentous, bursal or other pathology.

**Data analysis:** data was analyzed using SPSS version 20. The main outcome was abnormal findings seen on ankle ultrasound and the characteristics of these findings were described. The abnormalities were then categorized as tendon, joint, ligamentous, bursal or other pathology. A descriptive analysis of each variable in the data was conducted. Mean, proportions and standard deviation were used to describe the data. The data is presented in the form of frequency tables and bar graphs.

## Results

**Figure 1:**
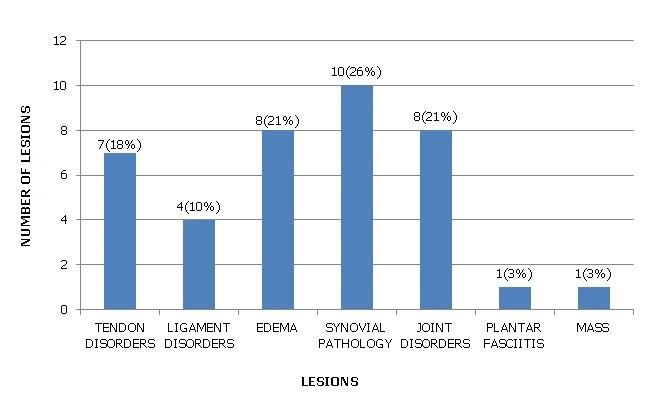
the distribution of lesions seen

**Figure 2:**
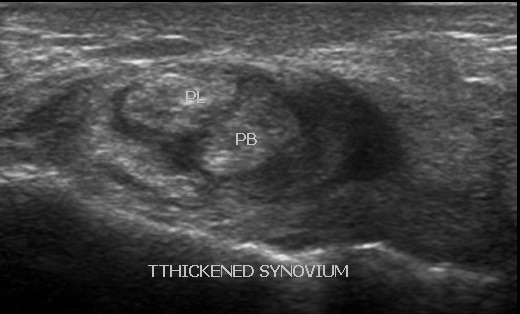
synovial hypertrophy 59 year old female patient with rheumatoid arthritis presented with ankle pain and swelling. Transverse ultrasound of the lateral compartment shows heterogeneously echogenic, non displaceable thickened synovium of the peroneal tendons

**Figure 3:**
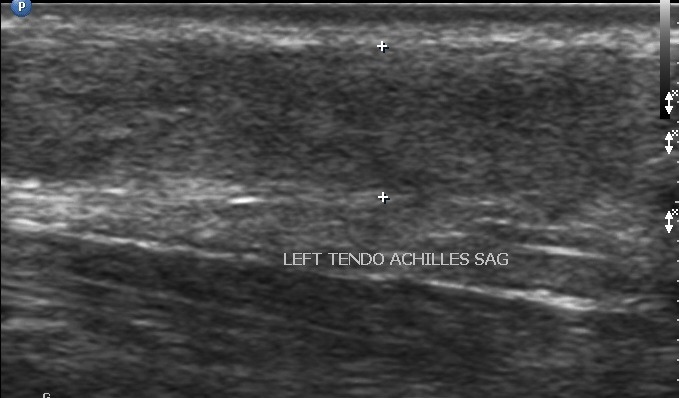
achilles tendinopathy 49 year old female presented with ankle swelling and heel pain for one week. Longitudinal ultrasound of the Achilles tendon shows an enlarged thickened tendon with loss of the normal fibrillary echo-texture

**Figure 4:**
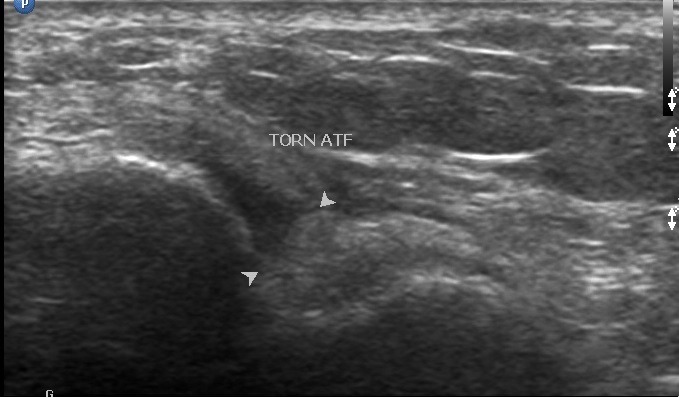
anterior talofibular ligament tear, 21 year old female patient with history of an ankle sprain. Ultrasound of the lateral compartment demonstrates a discontinuous ligament (arrow heads) with loss of attachment to the talus replaced by echogenic tissue. (ATF=Anterior talofibular ligament)

A total of 43 patients, 15(35%) males and 28(65%) females were examined. The mean age of the patients was 42.4±16.3 years with a median age of 39 years. The age range was between 18 and 67 years. Eleven (25.6%) of the patients presented with acute symptoms, while 32(74.4%) presented with chronic symptoms. The common presenting symptoms were ankle pain, ankle swelling, heel pain and snapping sound in the posterior compartment. Ankle pain was the most common presenting symptom and was present in 35 patients(81%); ankle swelling in 24 patients(56%); heel pain in 5 patients(12%) and snapping sound in 1 patient(2%). 20 patients(47%) presented with both ankle pain and swelling. The average duration of symptoms was 14.42 months in ankle pain; 10.96 months in ankle swelling; 5.79 months in heel pain and one day in snapping sound. Of the 43 patients, 12(28%) had a history of prior trauma; 2(5%) were athletes; 3(7%) had a history of quinolone use; 12(28%) had a co-morbid condition. The most common co-morbid condition was rheumatoid arthritis found in 6(50%) of the 12 patients who had co-morbid conditions; 1(8%) systemic lupus erythromatosus; 1(8%) systemic sclerosis; 1(8%) diabetes; 2(17%) osteoarthritis and 1(8%) fibromyalgia. Abnormalities were found in 26(60%) of the examinations done. A total of 39 lesions were identified (Figure 1). 25(64.1%) female patients were affected with lesions as compared to 14(35.9%) male patients. Synovial pathology was the commonest lesion seen and represented 26% of the lesions identified. All of the patients with synovial pathology were diagnosed with having synovial hypertrophy. 50% of the lesions were seen in patients with a history of rheumatoid arthritis (Figure 2). The commonest joint affected was the tarso-metatarsal joint seen in 5(50%) of the patients. Tendon disorders accounted for 19% of the lesions identified. Tendinopathy was the most prevalent pathology demonstrated (Figure 3). The Achilles tendon was the most common tendon involved seen in 4(57.1%) of patients. The flexor digitorum longus tendon was involved in 2(28.6%). Ligament disorders represented 4(11%) of the lesions identified. All lesions identified were anterior talofibular ligament tears (Figure 4). The tears were all found in female patients. Joint disorders accounted for 21% of the lesions seen (Table 1). The common abnormality identified was joint effusion and joint erosions. Edema represented 21% of the lesions identified. One patient was found to have plantar fasciitis which constituted 3% of the lesions seen. A mass was also seen in 1(3%) patient. The lesion did not fit any imaging criteria for a diagnosis of a specific condition

**Table 1:** spectrum of joint disorders

	Frequency	Percentage (%)
Joint Effusion		
Tibiotalar joint	3	37.5
Joint Erosions		
Metatarsophalengeal joints	2	25
Tarsometatarsal joints	1	12.5
Talonavicular osteoarthritis	1	12.5
Osteochondral defect	1	12.5

## Discussion

The ankle joint is the most commonly injured joint in the body. Although underutilized, musculoskeletal ultrasound is a cheap, easily available imaging modality with comparable sensitivity and specificity to MRI. This would be highly applicable in a resource poor setting. The examinations were performed in 28(65%) female patients and 15(35%) male patients. The mean age of the patients was 42.4 years. Similar findings were demonstrated by Artul S *et al* [15]. The most common symptom was ankle pain seen in 35(81%) patients. The second most common symptom was ankle swelling seen in 24(56%). The same was shown in Shalaby MH *et al.'s* study [16]. Synovial hypertrophy was the most prevalent pathology identified accounting for 26% of the lesions. This was higher than in studies by Artul S *et al*. [14] and Shalaby MH *et al*. [16] where the prevalence was 3% and 10.7% respectively. This could be explained by the fact that majority of the patients referred to us for ultrasound were from the rheumatology clinic. Tendon pathology was seen in 7(18%) of the patients examined. The most common tendon affected was the Achilles tendon representing 57.2% of the tendon lesions. The most common pathology demonstrated was tendinopathy accounting for 57.2% of the tendon pathologies seen. The anterior talofibular ligament was the ligament injured in all the ligamentous injuries seen. 75% represented partial tears. These findings are similar to the ones found in N. El-Liethy *et al* [10]. The major difference was the increased involvement of the FDL tendon in our study. Joint erosions and joint effusion were seen in association with other pathologies. Plantar fasciitis was seen in one case representing 3% of the lesions. This was lower than what was found by Shalaby MH *et al.* [16] and Artul S *et al*. [15]. Most patients with heel pain are referred for a radiograph, not ultrasonography. This would explain the discrepancy. In this study one case accounting for 3% of the lesions was diagnosed to have a mass. The lesion did not fit any particular imaging criteria for a diagnosis of a specific condition.

## Conclusion

A large spectrum of abnormalities was demonstrated with ultrasound in this study. This has shown the utility of ultrasound as a useful imaging modality when evaluating patients with ankle pain. This is especially so in a resource limited setting where expensive cross-sectional methods are not readily available. In view of specificity, sensitivity and ability to demonstrate a large spectrum of abnormalities, ultrasound should be recommended as the initial imaging modality in patients with ankle pain.

### What is known about this topic

- The ankle joint is the most frequently injured joint and is affected by traumatic injuries, inflammatory conditions, instability and osteoarthritis;
- Evaluation of the ankle joint for patients with ankle pain is initially performed using radiography and thereafter magnetic resonance imaging, for soft tissue evaluation;
- Ultrasound is a safe, cost effective imaging modality and has been shown to evaluate superficial structures with good resolution.

### What this study adds

- Ankle ultrasound was able to demonstrate a large spectrum of abnormalities;
- 60% of the examinations performed in patients with ankle pain were abnormal;
- The most common abnormality identified was synovial hypertrophy seen in 26% of the examinations.

## Competing interests

The authors declare no competing interests.

## References

[1] Nazarian L (2008). The top 10 reasons musculoskeletal sonography is an important complementary or alternative technique to MRI. AJR.

[2] Sharpe R, Nazarian L, Parker L (2012). Dramatically Increased Musculoskeletal Ultrasound Utilization From 2000 to 2009, Especially by Podiatrists in Private Offices. J Am Coll Radiol.

[3] Jacobson J (2009). Musculoskeletal Ultrasound: focused impact on MRI. AJR.

[4] Sconfienza L, Orlandi D, Lacelli F (2015). Dynamic High Resolution US of Ankle and Midfoot Ligaments: Normal Anatomic Structure and Imaging Technique. Radiographics.

[5] Neill J (2008). Musculoskeletal Ultrasound Anatomy and Technique.

[6] Mansour R, Jibri Z, Kamath S (2011). Persistent Ankle Pain Following a Sprain: a review of imaging. Emerg Radiol.

[7] Doherty C, Delahunt E, Caulfield B (2014). The incidence and prevalance of ankle sprain injury: a systematic review and meta-analysis of prospective epidemiological studies. Sports Med.

[8] Evans M, Schulany W (2006). Radiological Evaluation of High Ankle Sprain. Proc Bayl Univ Med Centre.

[9] Glover L (2014). Why Does an MRI cost so Darn Much?.

[10] El-Liethy N, Kamal H (2016). High resolution ultrasonography and magnetic resonance imaging in the evaluation of tendino-ligamentous injuries around ankle joint. The Egyptian Journal of Radiology and Nuclear Medicine.

[11] Polzer H, Kanz K, Prall W (2012). Diagnosis and Treatment of Acute Ankle Injuries: Development of an evidence based algorithm. Orthop Rev Pavia.

[12] Rockett M, Waitches G, Sudakoff G (1998). Use of Ultrasonography versus Magnetic Resonance Imaging for tendon abnormalities around the ankle. Foot Ankle Intl.

[13] Grant T, Kelikian A, Jereb S (2005). Ultrasound diagnosis of peroneal tendon tears: a surgical correlation. J Bone Joint Surg AM.

[14] Freidrich J, Schnarkowski P, Rubenacker S (1993). Ultrasonography of capsular morphology in normal and traumatic ankle joints. J Clin Ultrasound.

[15] Artul S, Habib G (2014). Ultrasound Findings of the Painful Ankle and Foot. J Clin Imaging Sci.

[16] Shalaby M, Sharara S, Abdelbary M (2017). High Resolution Ultrasonography in Ankle Joint Pain: Where does it Stand. Egypt J Radiol Nucl Med.

